# Supplementary material for: Identification of photoperiod-induced specific miRNAs in the adrenal glands of Sunite sheep (Ovis aries)
Source: Front Vet Sci. 2022 Jul 22;9:888207. doi: 10.3389/fvets.2022.888207 (PMC9354845; doi:10.3389/fvets.2022.888207)
Supplement: Supplementary file 1 [file Data_Sheet_1.ZIP › Supplementary file/miRDeep2 file.zip!/miRDeep2 file/Novel_Structure/LP42A1/Novel_260.pdf]

Provisional ID	:	NC_019460.2_7197
Score total	:	2.3
Score for star read(s)	:	-1.3
Score for read counts	:	0
Score for mfe	:	2
Score for randfold	:	1.6
Score for cons. seed	:	
Total read count	:	103
Mature read count	:	103
Loop read count	:	0
Star read count	:	0

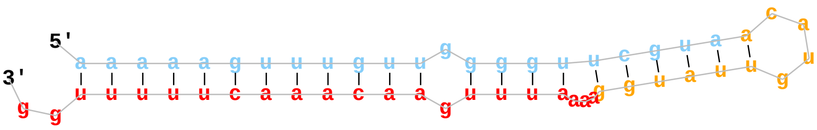

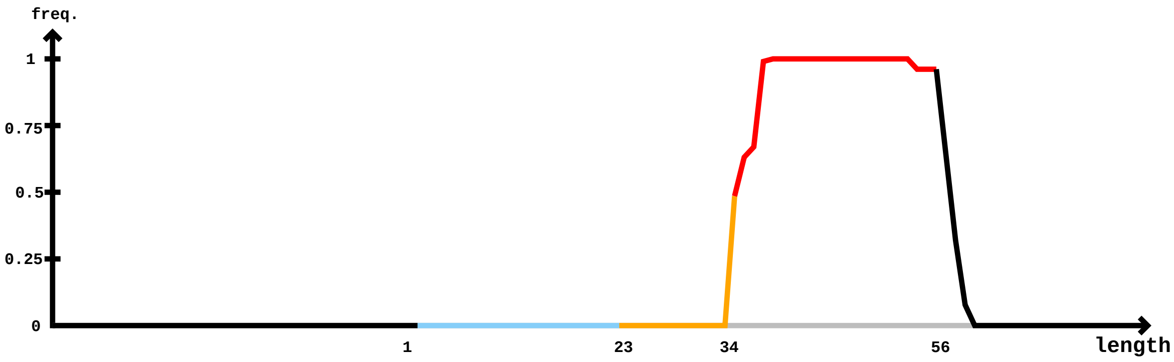

Star   Mature

```
5'- accacacucuggaaaucaguaaauauugguuggucaaaaaguuuguugggguucguaacauguuauggaaaauuugaacaaacuuuuuggccaacccaguauuauaaacca   -3'    exp
    .(((.....)))........(((((((((((((((((((((((((((.((((((((((....))))))...)))).)))))))))))))))))))))))))))........    reads    mm    sample
    ....................................................................aaaauCugaacaaacuuuu........................    4        1     seq
    ....................................................................aaaauCugaacaaacuuuuugg.....................    45       1     seq
    ....................................................................aaaauCugaacaaacuuuuuggc....................    1        1     seq
    .....................................................................aaauCugaacaaacuuuuugg.....................    14       1     seq
    .....................................................................aaauuugaacaaacuuuuuggc....................    1        0     seq
    ......................................................................aauCugaacaaacuuuuuggc....................    4        1     seq
    .......................................................................auCugaacaaacuuuuuggcc...................    25       1     seq
    .......................................................................auCugaacaaacuuuuuggcca..................    8        1     seq
    ........................................................................uCugaacaaacuuuuuggc....................    1        1     seq
```
